# Prostate motion during radiotherapy of prostate cancer patients with and without application of a hydrogel spacer: a comparative study

**DOI:** 10.1186/s13014-015-0526-1

**Published:** 2015-10-24

**Authors:** Prabhjot Juneja, Andrew Kneebone, Jeremy T. Booth, David I. Thwaites, Ramandeep Kaur, Emma Colvill, Jin A. Ng, Paul J. Keall, Thomas Eade

**Affiliations:** Northern Sydney Cancer Centre, Royal North Shore Hospital, Sydney, NSW 2065 Australia; Institute of Medical Physics, School of Physics, University of Sydney, Sydney, NSW 2006 Australia; 5/161A Willoughby Road, Naremburn, NSW 2065 Australia; Radiation Physics Laboratory, School of Medicine, University of Sydney, Sydney, NSW 2006 Australia

**Keywords:** Prostate cancer, Radiotherapy, Intrafraction motion, Hydrogel spacer

## Abstract

**Background and purpose:**

The use of a tissue expander (hydrogel) for sparing of the rectum from increased irradiation during prostate radiotherapy is becoming popular. The goal of this study is to investigate the effect of a tissue expander (hydrogel) on the intrafraction prostate motion during radiotherapy.

**Methods and material:**

Real time prostate motion was analysed for 26 patients and 742 fractions; 12 patients with and 14 patients without hydrogel (SpaceOAR™). The intra-fraction motion was quantified and compared between the two groups.

**Results:**

The average (±standard deviation) of the mean motion during the treatment for patients with and without hydrogel was 1.5 (±0.8 mm) and 1.1 (±0.9 mm) respectively (p < 0.05). The average time of motion >3 mm for patients with and without hydrogel was 7.7 % (±1.1 %) and 4.5 % (±0.9 %) respectively (p > 0.05). The hydrogel age, fraction number and treatment time were found to have no effect (*R*^*2*^ < 0.05) on the prostate motion.

**Conclusions:**

Differences in intrafraction motion in patients with hydrogel and without hydrogel were within measurement uncertainty (<1 mm). This result confirms that the addition of a spacer does not negate the need for intrafraction motion management if clinically indicated.

## Background

Daily image guidance correcting for interfraction motion, combined with intensity modulated/volumetric arc radiotherapy is considered the standard of care for prostate cancer radiotherapy in many centres [1, 2]. The use of a tissue spacer to move the rectum out of the high dose field was shown feasible initially with hyaluronic acid and collagen [3, 4] and now with the commercial availability of a polyethylene-glycol hydrogel absorbable water spacer (SpaceOAR™, Augmenix Inc., Waltham, MA) there has been more widespread utilisation. As radiotherapy protocols have moved to higher radiation doses and ultra hypofractionation (>6 Gy per fraction), smaller PTV margins are being used [5]. Initial results from daily interfraction correction using fiducial markers and a 3 mm posterior margin were concerning for poor biochemical control [6], raising the question of intrafraction motion.

Recently, studies have started to utilise intrafraction motion tracking techniques using Calypso [7] and kilovoltage intrafraction monitoring (KIM) [8] to mitigate the effect of prostate motion during treatment delivery. However, the effect of hydrogel on the prostate motion is not known, whether the prostate is more stable or less with hydrogel in place. The application of hydrogel pushes the prostate towards the fascia, to increase the distance between the prostate and the anterior rectal wall. The increased mass is hypothesised to assert lateral pressure that may stabilise the prostate, buffer from rectal wall movement; or conversely it may irritate the rectal wall causing discomfort and potentially more motion. It is clinically important to understand the effect of a hydrogel spacer on the prostate motion in order to have an evidence based assessment of the need for intrafraction monitoring, where applicable, or to quantify the magnitude of the PTV expansion required. This is important also because intrafraction monitoring can require expensive additional systems and cause stress to treatment staff and patient and increase the appointment times. To the best of the authors’ knowledge, the effect of hydrogel on intrafraction prostate motion has so far not been investigated.

Patients in our department enrolled in intrafraction motion monitoring studies [7, 8] comprised of two patient populations; those with and those without hydrogel spacer. The datasets from these studies provide a unique opportunity to investigate the impact of the hydrogel spacer on prostate motion. The aim of this study was to quantify the impact of hydrogel on intrafraction motion.

## Material and methods

### Patient data

After institutional review board (RNSH) approval, patients with informed consent were enrolled in 2 prospective clinical trials investigating novel techniques for intrafraction motion correction [7, 8]. In total 26 patients were available for analysis. Sixteen patients (503 fractions) were from the world’s first real-time dynamic multi-leaf collimator (MLC) tracking trial and were monitored using the Calypso® tracking system [7]. Another 10 patients (239 fractions) were from the world’s first kilo-voltage intra-fraction monitoring (KIM) trial and were monitored using kV fluoroscopy imaging [8]. Radiotherapy was delivered using a standard departmental protocol [9] with all patients simulated with an empty rectum and a comfortably full bladder. There were 12 patients (364 fractions) with hydrogel and 14 patients (378 fractions) without hydrogel.

### Hydrogel spacer implant

Hydrogel was inserted by one clinician (TE) at the time of fiducial marker or Calypso beacon implantation, using a transperineal approach as previously described [10].

### Treatment imaging

In the case of patients in MLC tracking trial, the real-time position of the prostate was measured using the Calypso® tracking system [7]. This uses implanted electromagnetic transponder-based markers [11]. Each patient had three electromagnetic markers implanted in the prostate gland. For the patients in the KIM trial, kV fluoroscopy was used to monitor, in real-time, the 3D position of three radio-opaque markers implanted into the prostate target [8, 12]. In both the trials, the location of the centroid of the implanted markers throughout treatment was used to calculate the intra-fraction prostate motion.

### Measure of prostate motion

Prostate motion was calculated using the probability of vector displacement. This plot provides a descriptive distribution of probabilities of various motion ranges [13]. The probability of vector displacement provides a detailed representation of the motion. For each prostate motion trajectory, motion was quantified by the mean of the largest x % of the vector displacements; x was investigated for 10, 20, 30, 40 and 50 %. For simplicity of presentation only results for 20 and 50 % are reported. Also, a mean of the vector displacements during the fractions was calculated. Previously in the literature, Langen et al. [14] have defined and used the fraction of time the prostate displacements are >3, >5, >7, and >10 mm; and Haisen et al. [15] have defined and investigated *R*_*95*_, which represents that the vector displacement is less than *R*_*95*_ during 95 percentage of the monitoring time. These metrics were also evaluated.

### Analysis

The probability of vector displacements for patients with and without hydrogel spacer was compared. Box plots were used to present and compare the differences in the motion metrics between these two groups. The Wilcoxon rank sum test was used as a test of the statistical significance of the differences and the Bonferroni correction [16] was used to counteract the significance threshold for multiple comparisons. Where several hypotheses are tested, the chance of obtaining at least one “statistically significant” result increases (even if all hypotheses are true) and therefore a correction method is needed to control for false positives. In addition, the effect of hydrogel age on prostate motion was also investigated.

As a secondary analysis, other factors that might affect prostate motion were also studied. The impact of treatment time and fraction number on motion was undertaken. The correlations were evaluated through the Pearson’s correlation, and coefficients of determination (*R*^*2*^) have been reported. These were calculated for all the available fractions (including patient with and without hydrogel), and for each patient, over their fractions. The difference between the prostate position at the start and end of the treatment has been used previously to describe intrafraction motion [17–19]. This hypothesis that the intra-fraction prostate motion range is equivalent to the difference between the prostate position at the start and end of the treatment was investigated using the Bland-Altman analysis [20]. The Bland-Altman analysis is a method to evaluate agreement between two measurement techniques. The prostate motion range of a fraction was defined by the maximum displacement between any two time points during the fraction.

## Results

**Fig. 1:**
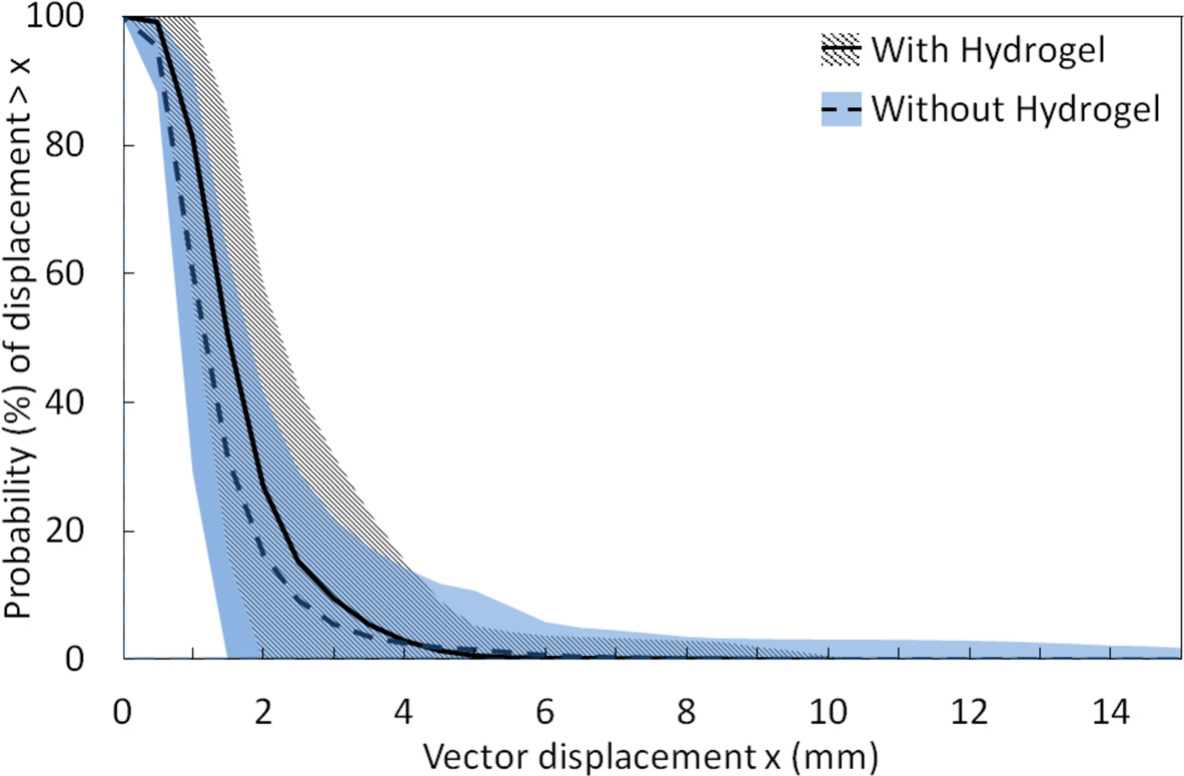
Probability of prostate motion (vector displacement) in the groups with hydrogel and without hydrogel. The central line is the mean probability. The shaded region shows mean ± 1 standard deviation

**Fig. 2:**
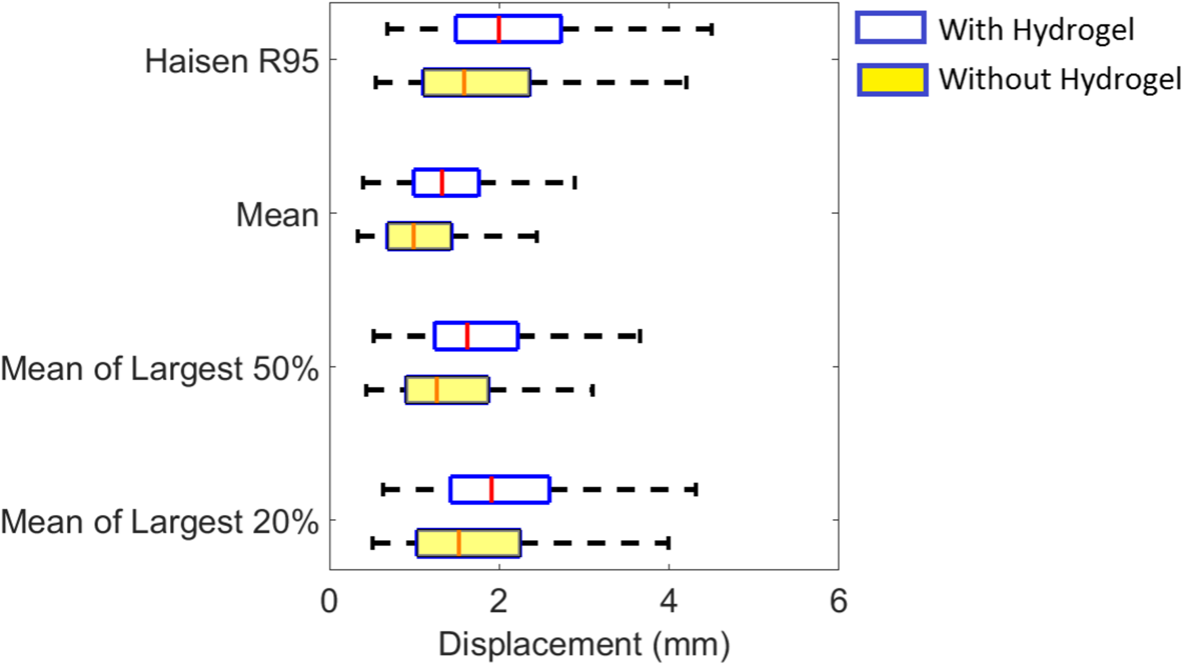
Comparison of vector displacement metrics in the groups with hydrogel and without hydrogel. On each box, the central mark is the median, the edges of the box are the 25th and 75th percentiles, the whiskers extend to maximum ± 2.7 standard deviation (~99 % coverage)

The probability of prostate motion (vector displacement) for patients with and without hydrogel is shown in Fig. 1. For the displacements less than 4 mm, corresponding mean probabilities of prostate motion are slightly higher for patients with hydrogel than without hydrogel. Another thing to note in Fig. 1 is that for larger displacement (>10 mm), the data is sparse (less than 5 non-zero values per data point) and therefore any conclusion could not be drawn regarding the effect of hydrogel in this motion range. The quantification of the motion using the predefined motion metrics is shown in Fig. 2. The results for largest 10, 20, 30, 40 and 50 % motion measures were similar. As mentioned earlier, for simplicity, results for 20 and 50 % only are reported. In the calculation of displacement greater than 3, 5, 7 and 10 mm metrics only the >3 mm threshold had more than 30 fractions in each of the groups with non-zero values. As such the other three >5 mm, >7 mm, and >10 mm were not considered further. The mean values (±standard deviation) of Langen >3 mm for patients with and without hydrogel were 7.7 % (±1.1 %) and 4.5 % (±0.9 %) respectively (*p* > 0.05). For all the metrics used in this study, except Langen’s, the motion was significantly higher (*p* < 0.05) in patients with hydrogel, however it should be noted the mean absolute differences were less than 0.5 mm which is similar to the measurement uncertainty.

### Effect of hydrogel age, fraction number, treatment time on prostate motion

**Fig. 3:**
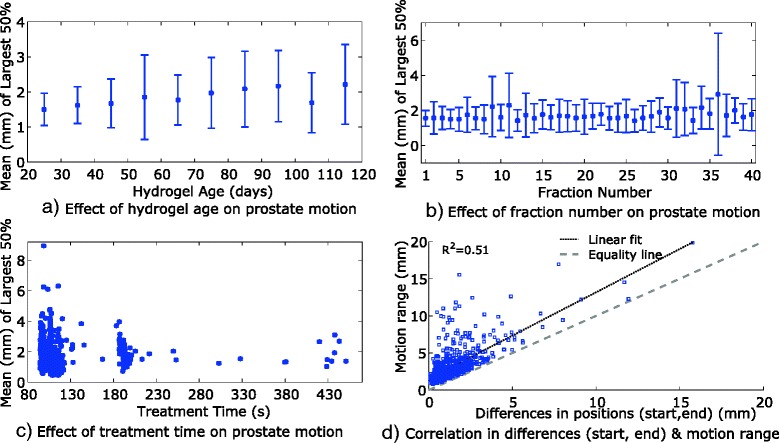
Correlation between prostate motion, mean (mm) of largest 50 % motion, and **a**. hydrogel age; **b**. fraction number; and **c**. treatment time. **d**. Correlation between differences in treatment start and end positions and prostate motion range. Note: Error bars in **a**. and **b**. represents mean ± standard deviation of all available fractions

No correlation (*R*^*2*^ < 0.05) was found between hydrogel age and prostate motion, for each of the motion metrics, across all the available fractions. The distribution of a motion metric, mean of the largest 50 % with respect to hydrogel age is presented in Fig. 3a. Furthermore, when analysis was performed for individual patients’ fractions, for each of the motion metrics, correlation was weak (*R*^*2*^ < 0.20). Similarly, fraction number and treatment time were found to be not correlated (*R*^*2*^ < 0.05) to the prostate motion, see Fig. 3b and c.

### Relationship between prostate motion range and difference in treatment start and end positions

The motion metrics, mean of the largest 20 % and 50 %, and Haisen R_95_, had moderate correlation (*R*^*2*^ ≈ 0.50) with the difference in prostate positions between start and end of the treatment. The prostate motion range was also found to have moderate correlation (*R*^*2*^ ≈ 0.51) with the difference in prostate positions between start and end of the treatment, see Fig. 3d. In the case of analysis of individual patients’ fractions, mean (range) correlation was 0.51 (0.05–0.91). The Bland-Altman analysis showed that the difference between the motion range and the differences in start and end positions were significantly different (*p* < 0.0001), the mean difference (range) was 1.7 mm (0.1–13.7 mm).

## Discussion

This study investigated for the first time the effect of hydrogel spacer on intrafraction prostate motion throughout the course of treatment. The mean differences between the motion metrics in the patient groups with and without hydrogel were found to be of the order of measurement uncertainty (≈0.5 mm) though if anything, greater intrafraction motion was seen in the hydrogel group. In line with this finding, Pinkawa et al. [21] have recently shown that inter-fraction prostate position variability is similar in patients who are treated with and without the hydrogel, with the motion modestly higher in the hydrogel group. It should be noted that their study used only two time points, one at the planning of treatment and another one in the last week of treatment while our study analysed real-time prostate motion data over the entire course of treatments.

This study has shown that there is minimal clinical difference in intrafraction motion in patients with and without hydrogel spacer. Furthermore, the age of the hydrogel spacer along with fraction number and treatment time were found to have no effect on the intra-fraction prostate motion (*R*^*2*^ < 0.05). This may partly be due to the low intrafraction motion in both groups with over 95 % of the fractions had mean motion less than 3 mm.

All of our patients were simulated with an empty rectum and comfortably full bladder but not specifically placed on a low residue diet. Intermittent cone beam CT was also used during treatment to aid the treatment staff with feedback to patients regarding bladder and rectal size. All patients were treated with a volumetric arc technique, which reflects beam on times more representative of a modern prostate cancer cohort, as compared to the original intrafraction motion data [14] that used a step and shoot technology. This can be seen in Fig. 3c. where the majority of the treatment beam on times were less than 3 min. Interestingly even in our patients with longer beam on times (>3 min, *n* = 74) due to hypofractionation, intrafraction motion was still low (mean motion: 0.5–2.7 mm).

Figure 3 also shows a comparison of continuous real-time tracking and a surrogate of intrafraction motion using the start and end of RT marker positions. This later technique has been used previously to describe intrafraction motion [17–19]. Although pre and post treatment displacement can give some information of the intra-fraction motion, our comparison shows this is not adequate. This was also shown by Noel et al. [22] using a Calypso® system. This technique commonly under-represents motion and in some cases can over-represent it. In the first case the prostate may have moved away from the initial setup for most of the treatment but near the end of RT returned closer to the pre RT position. In this case the cumulative dose impact will be significantly greater than the pre and post position data shows. The opposite may also occur, although less commonly, when the prostate is relatively stable for most of the treatment, but moves near the end of the beam on.

This study also found that the fraction number and treatment time have no effect on the intra-fraction prostate motion (*R*^*2*^ < 0.05). Kotte et al. [23] also did not find correlation between the intra-fraction prostate motion and the fraction number. Langen et al. [14] in their study compared the prostate motions in the first and last five treatment fractions of the patients and found non-significant differences. All the available fractions, with and without hydrogel were used in our analysis while these past studies only had patients without hydrogel. Previously, correlation between prostate motion and observation time (>5 min) has been shown [13, 14, 23, 24]. It should be noted that these studies considered intensity modulated radiotherapy (IMRT) and that the total time was not just the ‘actual’ treatment time (i.e. the start of first field to the end of last field) but also included the time between setup and beam on [13, 14, 24]. In comparison, the patients in the current study were treated with volumetric arc therapy and the observation time used was the ‘actual’ treatment time.

A study of prostate IMRT for a 2 Gy fraction has reported actual treatment times between 3.8 and 5.9 min depending on the number of fields (5–9 fields) [25]. In the current study, the analysis of fractions (*n* = 71) with hydrogel and treatment time greater than 3 min (3.06–7.52 min) found that their motion was not significantly different (*p* > 0.05) from the motion in fractions with times less than 3 min. Also, in these large fractions, the correlations between the motion metrics and treatment time was very low (*R*^*2*^ < 0.05).

Intrafraction motion is thought to be predominantly due to rectal motion, bladder filling, respiration, and/or patient movement [26]. It was unknown previously what impact a water based tissue expander (hydrogel) would have on motion, with the potential it could be decreased due to “fixation” of the prostate because hydrogel pushes prostate towards the fascia, or increased due to rectal wall irritability in the presence of hydrogel. It is reassuring that our study has confirmed no detriment with hydrogel in this respect. It is also important for clinicians using hydrogel to continue to consider intrafraction motion.

## Conclusions

The effect of addition of a hydrogel spacer on intrafraction prostate motion was examined and shown to be within measurement uncertainty (<1 mm) and felt to be clinically insignificant. Therefore, the clinical need for intrafraction motion management should be evaluated independently, irrespective of hydrogel presence or not.
